# Urinary incontinence among urban and rural community dwelling older women: prevalence, risk factors and quality of life

**DOI:** 10.1186/s12889-019-6870-6

**Published:** 2019-06-13

**Authors:** Resshaya R. Murukesu, Devinder K. A. Singh, Suzana Shahar

**Affiliations:** Centre for Healthy Aging and Wellness, Faculty of Health Sciences, Universiti Kebangsaan Malaysia, Jalan Raja Muda Abdul Aziz, Kuala Lumpur Malaysia

**Keywords:** Urinary incontinence, Community dwelling, Women's health, Geriatric, Urogenital health, Risk factors

## Abstract

**Background:**

Urinary incontinence (UI) is known to be more prevalent among women and is associated with decline in quality of life. The aim of our study was to investigate the prevalence, risk factors of urinary incontinence and its impact on quality of life among community dwelling older women living in urban and rural populations.

**Methods:**

This study was conducted based on secondary data analysed from the third phase of the longitudinal study “Neuroprotective Model for Health Longevity among Malaysian Elderly” (LRGS TUA). Stratification of urban and rural study areas were in accordance to that determined by the Department of Statistics. A total of 814 community dwelling older women (53% urban, 47% rural), aged 60 years and above, across four states within Peninsular Malaysia were included in this analysis. Interview-based questionnaires were used to obtain respondents’ sociodemographic details and clinical characteristics. The Timed Up and Go test and Handgrip Strength tests were used to assess physical function. Urinary incontinence was self-reported, and quality of life of those with incontinence was assessed using the King’s Health Questionnaire (KHQ).

**Results:**

Prevalence of urinary incontinence was 16% and 23% among older women living in urban and rural areas, respectively. Ethnicity was significantly associated with incontinence among older women in both urban and rural population (*p* < 0.05). Chronic constipation, functional mobility and muscle strength were associated with UI in participants from rural setting (*p* < 0.05). Binary logistic regression analysis showed that risk of incontinence is lower among Chinese [OR 0.430, 95% C.I: 0.224–0.825, *p* = 0.011] compared to Malay older women living in urban population. Within the rural population, respondents with chronic constipation [OR: 3.384, 95% C.I: 1.556–7.360, *p* = 0.002] were found to be at a higher risk of UI. In terms of quality of life, respondents in rural areas experienced more role, physical, social, emotional limitations and sleep disturbance as compared to their urban counterparts (*p* < 0.05).

**Conclusion:**

UI is more prevalent and had a more profound impact on quality of health among older women in the rural setting. The risk factors of UI were ethnicity and chronic constipation among urban and rural older women respectively. It is important to provide holistic strategies in the prevention and management of UI among older women especially within the rural population.

## Background

The global ageing population is rapidly increasing. Sixty five percent of the older population are women and experience higher rates of morbidity and disability due to longer life expectancies [1]. Defined as an involuntary loss of urine, urinary incontinence (UI) is one of the main causes of poor health in old age and is often perceived as a ‘women’s health’ issue [2, 3]. With the global prevalence of UI ranging between 3 to 53% among community dwelling older women, this condition is often underreported and unaddressed [4]. This is due to the misconception that UI is a part of the natural ageing process, or due to embarrassment, fear and unawareness of effective treatment options [5].

The significant risk factors of UI among older women within general population include increasing age, pre-existing medical conditions, impaired cognition and decline in physical function [4, 6]. Older adults dealing with UI are prone to skin infections, sexual dysfunction, loss of self-esteem, dependency, depression, frailty, institutionalisation, increased caregiver burden and economic cost [4, 7–9]. Moreover, incontinence disrupts normality of daily living and has an impact on psychosocial health leading to a decline in quality of life [10].

In the Malaysian setting, an ‘urban’ area is defined as a combined population of 10,000 people or more, whereas a rural area is one with a population of less than 10,000 people. About 60% of the population in the urban setting is involved in non-agricultural pursuits, while in the rural area it is more to do with agriculture and natural resources [11]. Rural areas are synonymous to poor living environments, may have less adequate healthcare services and older women there experience poorer health with less pro-active treatment seeking behaviors as compared to their counterparts in urban areas [12, 13]. Socioeconomic levels which includes location of settlement have been identified as predictors of UI [14, 15].

Information regarding the differences in prevalence, risk factors and quality of life among incontinent community dwelling older women within urban and rural settings is limited. Our aim was to conduct a secondary analysis of our previous study to examine the UI prevalence, risk factors and impact on quality of life among community dwelling older women within urban and rural populations.

## Methods

This study is a secondary analysis of the third phase of the larger longitudinal study “Neuroprotective Model for Health Longevity among Malaysian Elderly” (LRGS TUA). This large-scale study was designed to investigate the role of cognitive decline and associated risk factors through a comprehensive multi-dimensional assessment among community dwelling older adults [16]. It involved community dwelling older adults aged 60 years and above (*n* = 1560). Participants’ recruitment, study methodology and baseline characteristics have been previously described in detail [16].

### Study population

Our present study included community dwelling older women who participated in phase 3 of LRGS TUA between February to September 2016, across four states in Peninsular Malaysia (*n* = 814). Those with severe hearing and/or visual impairments, severe cognitive impairments (Mini Mental State Examination (MMSE) Score = < 15) [16] and were unable to speak or understand English or local dialects were excluded. Categorization of participants into urban and rural population was done based on stratification of the participating districts for each state as determined by the Malaysian Department of Statistics [11]. Ethical approval (UKM 1.5.3.5/244/NN-060-2013) and informed written consent of the participants were obtained. Participants were informed that information obtained at the time of data collection could be used for future research and publication purposes with the assurance of anonymity.

### Sociodemographic and clinical characteristics

Sociodemographic data and clinical characteristics was obtained via structured interview. It included age, ethnicity, marital status, years of education and history of smoking. Body mass index (BMI) was calculated after obtaining height and weight. Clinical characteristics included multimorbidity (the presence of 2 or more chronic diseases [17], history of falls (sustained at least 1 fall in the past 12 months [18]), chronic constipation, and mild cognitive impairment (MCI) [19]. Physical performance of participants was objectively assessed. The ‘Timed Up and Go’ (TUG) test was used to measure functional mobility and balance, whereby the more time taken (in seconds) to complete the test indicated lower functional mobility and balance. The dominant hand grip strength (HGS) was performed to quantify muscle strength in kilograms using the Jamar Plus + Hand Dynamometer (SI instruments Pty Ltd., SA, Australia). Details of screening and outcome measures included in our study, except the assessment of UI has been described by Shahar et al. [16]. Data was collected by research assistants of allied health personnel who were trained together prior to the commencement of data collection.

### Urinary incontinence and quality of life assessment

Urinary incontinence was self-reported based on the response ‘yes’ if participants were experiencing ‘any involuntary leakage of urine’ [3]. Those with incontinence and without were categorised as ‘UI’ and ‘Non-UI’ respectively. Participants with UI completed the King’s Health Questionnaire (KHQ) which asssessed theseverity of UI and its impact on quality of life (QoL) [20]. The KHQ was administered in both English and Malay language based on the participants’ preference. The translated Malay version KHQ was administered after establishing validity and reliability (Cronbach’s Alpha =0.87). Part 1 of KHQ described participants’ general health and perception of incontinence. Part 2 evaluated the level of limitation experienced (if any) for each quality of life domain as enumerated:

#### Role Limitations

Limitations of daily activities such as routine tasks within the house-hold or outside. Physical: Limitations in executing or participating in physical or functional activities.

#### Social Limitations

Quality of relationships and interactions with others (family & friends) and limitation in participation of social activities.

#### Personal Relationships

Quality of relationship with sexual partner, sex life and effect on marriage (Not applicable to those who were unmarried or sexually inactive).

#### Emotions

Focuses on level of depression, anxiety, nervousness, loss of self-esteem and self-respect being experienced.

#### Sleep/Energy

Disturbance in sleep quality and sleep deprivation.

#### Severity Measures

Not to be confused with severity of incontinence, ‘severity measures’ refers to measures taken to manage incontinence such as usage of pads for leakage, restriction of fluid intake, changing under garments. It also assesses worry of unpleasant odour.

Part 3 of KHQ evaluated the severity of incontinence based on 9 different incontinence characteristics (frequency, nocturia, urgency, stress incontinence, nocturnal enuresis, post-voidal dribbling, intercourse incontinence, urinary tract infections, bladder pain). Each component of the KHQ was rated with a Likert-type scale (‘very good’ to ‘very poor’; or ‘not at all’ to ‘a lot’). Scores of parts 1 and 2 of the KHQ was quantified in percentages, whereas part 3 was documented as total score. Higher percentage or score indicated lower quality of life [20, 21].

### Statistical analysis

Descriptive analysis of sociodemographic and clinical characteristic was performed among those with incontinence, comparing those from rural and urban populations. After a univariate analysis, a binary logistic regression analysis was performed to determine the risk factors of incontinence within population strata, with UI (yes or no) as the dependant variable. Regression analysis was carried out separately for urban and rural population. The impact of UI on quality of life was analysed using independent sample t-test using population strata (urban/rural) as the dependant variable. Significance level was set at *p* < 0.05. The IBM Statistical Package for Social Science (SPSS) version 22 was used for analysis.

## Results

### General characteristics of participants

Table 1 describes the baseline characteristics of the participants of this study. A total of 814 older women (mean age: 71.7 ± 5.7 years) participated in this study, with 53 and 47% accounting for urban and rural backgrounds respectively. Averagely, older women from rural areas were older than those from urban areas (*p* < 0.01). Ethnically, the study population comprised of the three predominant Malaysian ethnicities: Malay (58.6%), who made up for majority of the rural population, whereas the Chinese (36.6%) and Indians (4.8%) were mostly of the urban population (*p* < 0.001). Urban older women were mostly married and of higher education background. In contrast, rural older women were majority unmarried, divorced or widowed and had equal to or less than 6 years of education (*p* < 0.001). Though body mass index (BMI) was not found to have statistical significance, mean BMI was observed to be higher within the urban population, falling in the ‘overweight’ category. Rural older women had lower physical performance as they took longer time to perform TUG test and had lower HGS scores, implying less than optimal mobility and balance, and weaker muscle strength (*p* < 0.001).

**Table 1:** Baseline sociodemographic characteristics of participants (*n* = 814)

Variable	Total N	Strata n (%)		*P*-Value
	814	Urban 431 (52.9)	Rural 383 (47.1)	
Age (years) (mean±s.d.)	71.7±5.7	71.2±5.4	72.4±6.1	0.003**^+^
Ethnicity				<0.001***^#^
Malay	477	151 (35.0)	326 (85.1)	
Chinese	298	244 (56.6)	54 (14.1)	
Indian	39	36 (8.4)	3 (0.8)	
Marital Status				<0.001***^#^
Married	387	229 (53.1)	158 (41.3)	
Unmarried/Widowed/Divorced	427	202 (46.9)	225 (58.7)	
Years of Education				<0.001***^#^
≤ 6 years	665	316 (73.3)	349 (91.1)	
> 6 years	149	115 (26.7)	34 (8.9)	
Smoking Habit				0.230^#^
Smoker	17	7 (1.6)	10 (2.6)	
Non/Past Smoker	797	424 (98.4)	373 (97.4)	
BMI (kg/m^2^) (mean±s.d.)	25.1±4.7	25.3±4.5	24.9±4.9	0.198^+^
Multimorbidity				0.320
< 2 chronic diseases	474	258 (59.9)	216 (56.4)	
≥ 2 chronic diseases	340	173 (40.1)	167 (43.6)	
Urinary Incontinence				0.008*^#^
No	657	362 (84.0)	295 (77.0)	
Yes	157	69 (16.0)	88 (23.0)	
History of Falls				0.125^#^
No	661	359 (83.3)	302 (78.9)	
Yes	144	701 (16.7)	74 (21.1)	
Chronic Constipation				0.101^#^
No	749	402 (93.3)	347 (90.6)	
Yes	65	29 (6.7)	36 (9.4)	
Mild Cognitive Impairment				0.116^#^
No	741	387 (89.8)	354 (92.4)	
Yes	73	44 (10.2)	29 (7.6)	
Physical Performance (mean±s.d)				
Functional Mobility (TUG) (sec)	12.2±4.0	11.2±3.6	13.3±4.2	<0.001***^+^
Muscle Strength (HGS) (kg)	18.9±4.8	20.6±4.8	17.5±4.4	<0.001***^+^

* *p* < 0.05, **p < 0.01, ***p < 0.001, ^#^ χ^2^ test was used,^+^Independent Samples T-Test was used

*BMI* Body Mass Index, *TUG* Timed Up and Go, *HGS* Handgrip Strength, *kg* Kilogram, *sec* seconds

### Prevalence of urinary incontinence (UI)

A total of 157 (19%) older women self-reported UI and was found to be higher among the rural population (*p* < 0.01) (Table 1). The findings of this study determined the prevalence of UI among urban and rural population to be 16 and 23%, respectively.

### Factors associated with UI within urban and rural populations

The association between variables and incontinence (UI/Non-UI) within urban and rural older women is as outlined in Tables 2 and 3 respectively. The Chi-square analysis indicated that ethnicity was significantly associated with incontinence among urban older women (*p*<0.05), as well as among rural older women (*p*<0.05). Within both populations, Malay older women made up majority of the UI groups (47.8% in urban, 88.6% in rural), followed by the Chinese and Indians. The direction of association is further explored in the regression analysis. Chronic constipation (47.2% UI with constipation; 20.5% UI without constipation, *p* < 0.001) and physical performance; functional mobility (*p*<0.05) and muscle strength (*p* < 0.001) were also found to be significant among rural older women. When compared to continent older women, those with UI took a longer time to complete the TUG test which implied impairment in mobility and dynamic balance. Similarly, older women with UI had lower handgrip strength implying less than optimal overall muscle strength.

**Table 2:** Association between variables and incontinence in urban population

Variable	Total	Urban n (%)		*P*-Value
	431	UI 69 (16.0)	Non-UI 362 (84.0)	
Age (years) (mean ± s.d.)	71.2 ± 5.4	71.1 ± 5.5	71.2 ± 5.4	0.812^+^
Age Range				0.678^#^
60–69	194	32 (46.4)	162 (44.8)	
70–79	199	32 (46.4)	167 (46.1)	
≥ 80	38	5 (7.2)	33 (9.1)	
Ethnicity				0.042*^#^
Malay	151	33 (47.8)	118 (32.6)	
Chinese	244	30 (43.5)	214 (59.1)	
Indian	36	6 (8.7)	30 (8.3)	
Marital Status				0.518^#^
Married	229	37 (53.6)	192 (53.0)	
Unmarried/Widowed/Divorced	202	32 (46.4)	170 (47.0)	
Education				0.637^#^
≤ 6 Years	316	49 (71.0)	267 (73.8)	
> 6 Years	115	20 (29.0)	95 (26.2)	
Smoking History				0.312^#^
Smoker	7	2 (2.9)	5 (1.4)	
Non/Past Smoker	424	67 (97.1)	86 (98.6)	
BMI (kg/m^2^) (mean ± s.d.)	25.3 ± 4.5	26.0 ± 4.5	25.2 ± 4.5	0.200^+^
Multimorbidity				0.091^#^
< 2 chronic diseases	258	35 (50.7)	223 (61.6)	
≥ 2 chronic diseases	173	34 (49.3)	139 (38.4)	
History of Falls				0.455^#^
No	359	57 (82.6)	302 (83.4)	
Yes	70	12 (17.4)	58 (16.6)	
Chronic Constipation				0.073^#^
No	402	61 (88.4)	341 (94.2)	
Yes	29	8 (11.6)	21 (5.8)	
Mild Cognitive Impairment				0.072^#^
No	387	58 (84.1)	329 (85.0)	
Yes	44	11 (15.9)	33 (15.0)	
Physical Performance (mean ± s.d)				
Functional Mobility (TUG) (seconds)	11.2 ± 3.6	11.9 ± 4.4	11.1 ± 3.3	0.059^+^
Muscle Strength (HGS) (kg)	20.1 ± 4.6	20.6 ± 4.8	19.9 ± 4.6	0.270^+^

* *p* < 0.05, ^#^ χ^2^ test was used, ^+^Independent Samples T-Test was used

*BMI* Body Mass Index, *TUG* Timed Up and Go, *HGS* Handgrip Strength, *kg* Kilogram, *sec* seconds

**Table 3:** Association between variables and incontinence in rural population

Variable	Total	Rural n (%)		*P*-Value
	383	UI 88 (23.0)	Non-UI 295 (77.0)	
Age (years) (mean ± s.d.)	72.4 ± 6.1	73.2 ± 6.6	72.2 ± 5.9	0.184^+^
Age Range				0.678^#^
60–69	145	30 (34.1)	115 (39.0)	
70–79	183	38 (43.2)	145 (49.2)	
≥ 80	55	20 (22.7)	35 (11.8)	
Ethnicity				0.039*^#^
Malay	326	78 (88.6)	248 (84.1)	
Chinese	54	9 (10.2)	45 (15.3)	
Indian	3	1 (1.1)	2 (0.7)	
Marital Status				0.458^#^
Married	158	26 (29.5)	132 (44.7)	
Unmarried/Widowed/Divorced	225	62 (70.5)	163 (55.3)	
Education				0.173^#^
≤ 6 Years	349	77 (87.5)	272 (92.2)	
> 6 Years	34	11 (12.5)	23 (7.8)	
Smoking History				0.312^#^
Smoker	10	2 (2.3)	8 (2.7)	
Non/Past Smoker	373	86 (97.7)	287 (97.3)	
BMI (kg/m^2^) (mean ± s.d.)	24.9 ± 4.9	25.2 ± 5.2	24.7 ± 4.8	0.428^+^
Multimorbidity				0.374
< 2 chronic diseases	216	46 (52.3)	170 (57.6)	
≥ 2 chronic diseases	167	42 (47.7)	125 (42.4)	
History of Falls				0.447^#^
No	302	69 (78.4)	233 (79.0)	
Yes	74	18 (21.6)	56 (21.0)	
Chronic Constipation				< 0.001***^#^
No	347	71 (80.7)	276 (93.6)	
Yes	36	17 (19.3)	19 (6.4)	
Mild Cognitive Impairment				0.516^#^
No	354	81 (92.0)	279 (94.6)	
Yes	29	7 (8.0)	22 (5.4)	
Physical Performance (mean ± s.d)				
Functional Mobility (TUG) (seconds)	13.3 ± 4.2	14.2 ± 5.4	13.0 ± 3.6	0.008**^+^
Muscle Strength (HGS) (kg)	18.6 ± 4.8	17.5 ± 4.4	19.0 ± 4.8	< 0.001***^+^

* *p* < 0.05, **p < 0.01, ***p < 0.001, ^#^ χ^2^ test was used, ^+^Independent Samples T-Test was used

*BMI* Body Mass Index, *TUG* Timed Up and Go, *HGS* Handgrip Strength, *kg* Kilogram, *sec* seconds

### Risk factors of UI within urban and rural populations

The regression analysis included variables that were significant in this study as well as those reported to be significant in past studies (age, ethnicity, education, BMI, morbidity, history of falls, chronic constipation, MCI and physical performance). Ethnicity was a non-binary, categorical variable. Malay ethnicity was selected as the reference variable as it is the largest ethnicity in Malaysia. Tables 4 and 5 depict the results of the binary logistic regression for urban and rural populations respectively. Results among the urban population found older women of Chinese ethnicity to be 0.6 times less likely to have UI as compared to Malay ethnicity [OR 0.430, 95% C.I: 0.224–0.825, *p* = 0.011]. In the rural setting, older women with chronic constipation [OR: 3.384, 95% C.I: 1.556–7.360, *p* = 0.002] were found to be at risk of UI by 3.4 times compared to those without constipation.

**Table 4:** Risk factors of UI within urban population

Risk Factor	Adj. OR ((%%CI)	*β*	*p*-value
Age	1.016 (0.956–1.080)	0.016	0.610
Ethnicity			0.040
Chinese	0.430 (0.224–0.825)	−0.845	0.011*
Indian	0.741 (0.242–2.267)	−0.299	0.600
Education	1.375 (0.700–2.700)	0.318	0.355
Body Mass Index	0.999 (0.936–1.066)	−0.001	0.976
Multimorbidity	1.710 (0.943–3.100)	0.536	0.077
History of Falls	1.274 (0.608–2.671)	0.242	0.521
Chronic Constipation	1.718 (0.638–4.624)	0.541	0.284
Mild Cognitive Impairment	0.639 (0.296–1.382)	− 0.447	0.255
Muscle Strength (HGS)	1.056 (0.989–1.127)	0.054	0.105
Mobility & Balance (TUG)	1.061 (0.977–1.153)	0.060	0.158

* *p* < 0.05

*TUG* Time Up and Go Test, *HGS* Hand Grip Strength

**Table 5:** Risk factors of UI within rural population

Risk Factor	Adj. OR ((%%CI)	*β*	*p*-value
Age	1.021 (0.968–1.076)	0.021	0.448
Ethnicity			0.045
Chinese	1.307 (0.429–3.987)	0.268	0.637
Indian	0.537 (0.185–1.778)	0.557	0.335
Education	1.911 (0.776–4.708)	0.648	0.159
Body Mass Index	1.036 (0.976–1.099)	0.035	0.243
Multimorbidity	1.140 (0.657–1.999)	0.131	0.647
History of Falls	0.935 (0.476–1.837)	−0.068	0.845
Chronic Constipation	3.384 (1.556–7.360)	1.219	0.002**
Mild Cognitive Impairment	0.939 (0.361–2.443)	−0.063	0.897
Muscle Strength (HGS)	0.947 (0.890–1.008)	−0.054	0.088
Mobility & Balance (TUG)	1.029 (0.959–1.105)	0.029	0.424

**p < 0.01

*TUG* Time Up and Go Test, *HGS* Hand Grip Strength

### Quality of life of older women within urban and rural populations

Table 6 depicts the quality of life of the urban and rural older women with incontinence based on the KHQ. Both urban and rural populations expressed positive general health perception in Part 1 (*p* < 0.05). However, 96% of the participants reported that daily life was impacted due to UI by at least ‘a little’. The findings in Part 2 of the KHQ showed that older women in the rural setting had higher scores for all the domains of QoL. Incontinent older women dealt with significantly higher role, physical and social limitations as well as emotional and sleep disturbances (*p* < 0.05). Part 3 found no significant difference between the two populations, implying that both rural and urban older women experienced similar levels of incontinence severity. Though the incontinence related symptom of ‘bladder pain’ was statistically significant with it affecting the urban population, the credibility of significance is questionable with the close to 0 mean score.

**Table 6:** Quality of life among participants with UI according to strata

Domains of KHQ	Total N	Strata n (%)		*P*-Value
	157	Urban 69 (43.9)	Rural 88 (56.1)	
Part 1 n (%)				
General Health Perception				0.040*^+^
Very Good	6	0	6 (6.8)	
Good	100	51 (73.9)	49 (55.7)	
Fair	46	16 (23.2)	30 (34.1)	
Poor	5	2 (2.9)	3 (3.4)	
Very Poor	0	0	0	
Incontinence Impact				0.204
Not at All	6	5 (7.2)	1 (1.1)	
A Little	80	36 (52.2)	44 (50.0)	
Moderately	67	26 (37.8)	41 (46.6)	
A Lot	4	2 (2.9)	2 (2.8)	
Part 2 (mean score ± s.d) (%)				
Role Limitation	38.7 ± 18.4	34.5 ± 17.9	42.0 ± 18.2	0.011*
Physical Limitation	50.8 ± 22.4	46.9 ± 21.6	54.0 ± 22.6	0.048*
Social Limitation	17.4 ± 22.6	12.1 ± 21.1	21.6 ± 22.9	0.008**
Personal Relationship	4.1 ± 11.7	3.1 ± 8.2	4.9 ± 13.7	0.346
Emotions	31.4 ± 27.5	25.3 ± 24.8	36.2 ± 28.6	0.013*
Sleep	46.2 ± 27.1	38.9 ± 24.9	51.9 ± 27.5	0.003**
Severity Measures	34.8 ± 21.7	32.4 ± 23.1	36.7 ± 20.6	0.212
Part 3 (mean score ± s.d)				
Overall Symptom Severity	8.6 ± 2.5	8.9 ± 2.6	8.3 ± 2.4	0.105
Frequency	2.2 ± 0.7	2.2 ± 0.7	2.2 ± 0.7	0.766
Nocturia	1.7 ± 1.5	1.8 ± 1.2	1.7 ± 1.2	0.699
Urge Incontinence	1.0 ± 0.9	1.1 ± 1.0	0.9 ± 0.8	0.087
Stress Incontinence	2.4 ± 0.6	2.3 ± 0.6	2.4 ± 0.6	0.500
Nocturnal Enuresis	0.8 ± 0.9	0.9 ± 0.9	0.8 ± 0.8	0.210
Post Voidal Dribbling	0.3 ± 0.7	0.4 ± 0.7	0.3 ± 0.7	0.433
Intercourse Incontinence	0.1 ± 0.3	0.1 ± 0.3	0.1 ± 0.2	0.339
Urinary Tract Infections	0.1 ± 0.2	0.1 ± 0.3	0.1 ± 0.2	0.473
Bladder Pain	0.1 ± 0.2	0.1 ± 0.3	0.0 ± 0.1	0.011*

* *p* < 0.05, **p < 0.01

## Discussion

To the best of our knowledge, this study is the first of its kind as it compares the prevalence, its associated risk factors and quality of life among Malaysian community dwelling older women within the urban and rural settings. Employing the definition recommended by the International Continence Society for prevalence studies, the prevalence of UI was reported to be higher among older women in the rural community setting [3].

Among older women in rural settings, prevalence of UI in our large-scale study (23%) is considered high compared to a smaller previous study (3%) which was conducted in one state with smaller number of participants [22]. This sizeable difference could be explained by the study design. Prevalence of UI in our study is between the 10% reported in India, 40% in Spain and 36% in the United States among rural populations [23–25].

In the urban population, the prevalence of UI in our study was reported at 16%. This is comparatively higher compared to Singapore (5%), but lower than Puerto Rico (35%) [26, 27]. There are limited studies reporting prevalence of UI among older women specific to urban or rural population. It was observed that prevalence reported in our current study and those from Asia were lower when compared to Western countries.

It could be due to normalising UI as a consequence of ageing and cultural sensitivity [28] resulting in underreporting of UI among older women in Asian rural settings. Lower socioeconomic background, poorer living conditions, inaccessibility or unawareness of healthcare services and dismissing incontinence as a health concern could have also accounted for higher prevalence of UI among older women in rural communities [12, 14, 15]. In addition, primary healthcare providers often overlook or omit asking UI related questions during health screenings or check-ups in rural settings [29].

Our study results showed different risk factors of UI among older women in urban and rural settings. Ethnicity was significantly associated with incontinence among older women in both urban and rural population (*p* < 0.05). However, ethnicity was found to be the only significant risk factor for urban older women. Urban older women of Chinese ethnicity were found to be 60% less likely to experience UI when compared to Malay older women. Incontinence and ethnic disparity has yet to be studied in detail in Malaysia. However, Malaysian Chinese have been found to be least likely to report poor health and had lower prevalence among non-communicable diseases when compared to the Malay and Indian populations [30]. A study in the United States examining prevalence of incontinence and its association to a particular ethnicity deduced that anatomical and physiological differences such as ‘lower urethral closure pressure’, may increase susceptibility of leakage [31]. This could potentially be the rationale behind our study’s findings as well. Future research may consider delving further into Asian ethnic disparity and likelihood of UI.

Although chronic constipation, functional mobility and muscle strength were associated with UI in older women residing in rural setting (*p* < 0.05), only chronic constipation appeared as a significant risk factor. Chronic constipation was found to increase the risk of incontinence by 3.4 times among older women in our study. Similarly, chronic constipation was reported to be a predictor of UI among women residing in a rural community in India [21]. This is in agreement with findings of past studies which correlated UI with the increase in intra-abdominal pressure among individuals with severe constipation [5, 32, 33].

Falling under the mnemonic ‘DIAPPERS’, chronic constipation due to stool impaction is a treatable cause of UI with the right medication and lifestyle modification [5]. Poor physical function namely functional mobility and strength has been strongly correlated with increased likelihood of incontinence, especially among older adults who are home-bound or living in institutions [34]. Decline in physical performance is associated with increased risk of falls and it hampers the ability to carry functional tasks which is a vital aspect of successful toileting [35, 36]. The participants in our study were community dwelling and considerably independent in terms of daily functionality, which could explain why physical function was not found to be a significant risk factor of UI.

The existence of urban-rural gradient among incontinent has been established whereby women from urban communities perceived incontinence to negatively impact quality of life and were more likely to seek treatment as compared to those in rural communities [37]. In our study, older women from both populations equally perceived impact of UI in general between little to moderate. In regard to specific domains of daily life, it impacted the rural population more. It is possible that older women from rural populations with lower education levels are ignorant or have less understanding of UI as a health condition. They probably perceived themselves to have decent wellbeing despite experiencing limitations in multiple facets of daily living due to incontinence [38, 39].

In our study, older women in the rural setting dealt with higher limitations in role, social and physical aspects of daily living due to incontinence. It could be inferred that inability to execute habitual tasks may cripple one’s sense of purpose, resulting in a loss of personal identity [40]. Social life plays an important role among rural non-institutionalised older adults and ‘social exclusion’ can be hazardous to the community [41, 42]. Maintaining strong ties within the community such as frequent social calls among friends and relatives are integral components of daily living in the rural setting and is less valued within urban populations [43]. It could be deduced that rural older women were more sociable than urban hence experiencing more impact on this domain when dealing with UI.

Incontinent older women in the rural population were also more emotionally disturbed, expressing higher levels of depression, anxiety and low self-esteem. Feelings of loneliness and depression have been found to be more prevalent among rural older adults living alone [40, 44]. This is more likely to increase with the presence of UI, fear of stigmatisation and embarrassment associating incontinence with emotional vulnerability [40, 44]. Quality of sleep was poorer among rural older women as they dealt with tiredness and sleep disturbances in sleep domain due to UI and it can be explained by the presence of nocturia and nocturnal enuresis. These two common symptoms of UI have been enlisted as causes of chronic sleep deprivation among those with UI [45].

The strength of our study is that we investigated incontinence among older women from both urban and rural settings. In addition, the participants were non-institutionalised and were ethnically diverse. The findings of our study can be used as a comparison with nations of similar, multi-ethnic population. However, the cross-sectional design of this study is only able to determine the risk factors but not examine the causal relationship of potential risk factors and UI. As UI is perceived to be a considerably sensitive health issue, the possibility of underreporting or self-reporting bias may have occurred. Objective assessment of UI was not administered due to non-feasibility of the test in this large-scale study. Also, treatment seeking behaviours for incontinence was not investigated which would be highly recommended for future studies. Clinically, addressing the modifiable risk factors prior to incontinence management could possibly improve the treatment outcome in both urban and rural settings.

## Conclusion

In conclusion, our study showed that UI is prevalent among community dwelling older women. However, its impact on quality of life was higher among older women in the rural population. Risk factors of UI were different in both urban and rural populations. This suggests that specifically tailored UI prevention and treatment strategies could be beneficial, especially in older women residing at rural areas.

## Abbreviations

BMI: Body mass index
CI: Confidence interval
HGS: Handgrip strength
KHQ: King’s Health Questionnaire
LRGS TUA: Neuroprotective Model for Health Longevity among Malaysian Elderly
MCI: Mild cognitive impairment
OR: Odds ratio
QoL: Quality of life
SPPS: Statistical package of social sciences
TUG: Timed Up and Go
UI: Urinary incontinence
